# Alteration in Cellular Signaling and Metabolic Reprogramming during Viral Infection

**DOI:** 10.1128/mBio.00635-21

**Published:** 2021-09-14

**Authors:** Anil Pant, Lara Dsouza, Zhilong Yang

**Affiliations:** a Division of Biology, Kansas State University, Manhattan, Kansas, USA; b Department of Veterinary Pathobiology, College of Veterinary Medicine & Biomedical Sciences, Texas A&M University, College Station, Texas, USA; Harvard Medical School, Brigham and Women's Hospital; Albert Einstein College of Medicine

**Keywords:** virus, metabolism, host-virus interactions, growth signaling, PI3K-Akt-mTOR, AMPK pathway, HIF-1α, metabolic disorders, HIF-1alpha, virus

## Abstract

Cellular activities are finely regulated by numerous signaling pathways to support specific functions of complex life processes. Viruses are obligate intracellular parasites. Each step of viral replication is ultimately governed by the interaction of a virus with its host cells. Because of the demands of viral replication, the nutritional needs of virus-infected cells differ from those of uninfected cells. To improve their chances of survival and replication, viruses have evolved to commandeer cellular processes, including cell metabolism, augmenting these processes to support their needs. This article summarizes recent findings regarding virus-induced alterations to major cellular metabolic pathways focusing on how viruses modulate various signaling cascades to induce these changes. We begin with a general introduction describing the role played by signaling pathways in cellular metabolism. We then discuss how different viruses target these signaling pathways to reprogram host metabolism to favor the viral needs. We highlight the gaps in understanding metabolism-related virus-host interactions and discuss how studying these changes will enhance our understanding of fundamental processes involved in metabolic regulation. Finally, we discuss the potential to harness these processes to combat viral diseases, as well as other diseases, including metabolic disorders and cancers.

## INTRODUCTION

Metabolism is fundamental to cell survival and function. Signal transduction is essential for the regulation and coordination of cell metabolism. Because viruses do not have their own metabolic capabilities, in order to replicate successfully, they must actively interact with and usurp key cellular signaling pathways to modulate cellular energy and nutrition metabolism. For example, human cytomegalovirus (HCMV) upregulates almost all aspects of cell metabolism to support productive infection (1). Vaccinia virus (VACV) increases the levels of tricarboxylic acid (TCA) cycle intermediates and glutamine metabolism to support highly efficient replication (2–4). Outstanding review articles about virus-induced alterations to the host metabolic process can be found elsewhere (5, 6).

**FIG 1:**
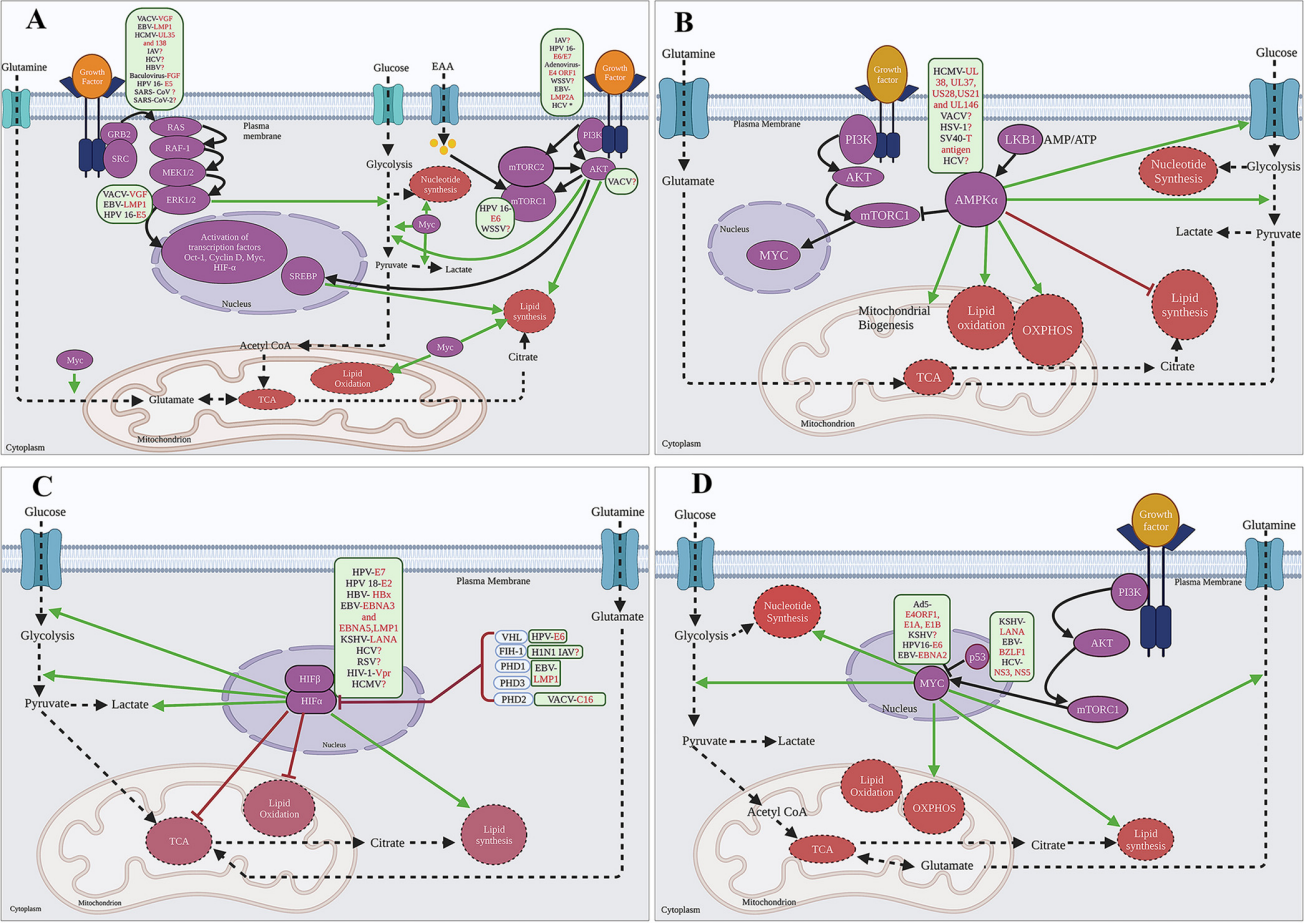
Viruses target cellular signaling pathways to alter host cell metabolism. Viruses (shown in green boxes) (their known viral factor/factors are highlighted in red) either directly or indirectly rewire the growth factor signaling pathways, RTK and PI3K-Akt pathway (A), AMPK pathway (B), HIF pathway (C), or the Myc oncogene and p53 tumor suppressor (D) to hijack cellular metabolism. The red question marks indicate that the viral factors responsible are still unknown. The viruses in the green boxes are placed right next to the signaling pathway intermediates or receptors they have been known to activate. The cellular signaling pathways are highlighted in purple. Activation of these signaling pathways directly or indirectly influence different metabolic pathways as highlighted in orange boxes. The solid black pointed arrows indicate activation of signaling pathway. The dashed black pointed arrows indicate flow of metabolites. The solid green pointed arrows indicate activation of metabolic pathways. The solid, blunt black or brown arrows indicate inhibition of a signaling intermediate or a reduction or inhibition of metabolic pathways. Ad5, adenovirus 5; EBV, Epstein-Barr virus; HCV, hepatitis C virus; HBV, hepatitis B virus; HSV-1, herpes simplex virus 1; HCMV, human cytomegalovirus; HIV, human immunodeficiency virus; HPV 16, human papillomavirus type 16; IAV, influenza virus; KSHV, Kaposi’s sarcoma-associated herpesvirus; RSV, respiratory syncytial virus; SARS-CoV, severe acute respiratory syndrome coronavirus; SARS-CoV-2, severe acute respiratory syndrome coronavirus 2; SV40, simian virus 40; VACV, vaccinia virus; WSSV, white spot syndrome virus. *HCV suppresses the PI3K-AKT pathway. The relative size of cellular organelles is not representative of the true relative size of these organelles in the cell. This figure was created in Biorender.com.

Viruses exploit several strategies to hijack cellular nutrient resources. Some viruses upregulate core catabolic processes (e.g., glycolysis and the TCA cycle), whereas others target anabolic and biosynthetic processes (e.g., nucleotide, fatty acid [FA], and amino acid synthesis) (6). These metabolic processes are governed by key cellular signaling cascades, including growth factor signaling, phosphoinositide 3-kinase (PI3K)-protein kinase B (Akt), and AMP-activated protein kinase (AMPK) pathways (7, 8). Unsurprisingly, viruses have evolved mechanisms that directly or indirectly target these pathways to generate a favorable metabolic environment to support viral replication. This article summarizes several cellular signaling pathways crucial for regulating metabolism and describes our current understanding of how viruses interact with these pathways to meet the increased demands for metabolites and energy necessary for replication (Fig. 1 and Table 1).

**TABLE 1:** Viruses regulate cellular signaling pathways to repurpose host cell metabolism^*a*^

Signaling pathway	Virus(es)	Virus protein(s)	Cellular target(s)	(Potential) metabolic effect(s)	Reference(s)
Growth factor signaling	VACV	VGF	EGFR (+), MAPK (+), pSTAT3 S727 (+)	TCA cycle (+)	24
	Baculovirus	vFGF	?	Glucose, glutamine uptake (+)	31
	EBV	LMP1	FGFR (+)	Glycolysis (+)	36, 37
	SARS-CoV	?	EGFR (+)	?	39
	SARS-CoV-2	?	EGFR (+)	Glycolysis (+), TCA cycle (+)	40, 41
PI3K-AKT- mTOR signaling	VACV	?	AKT (+)	Lipid metabolism (?)	3, 24, 59
	HPV 16	E6/E7	PI3K-AKT (+)	Glycolysis (+)	48, 64, 65
	WSSV	?	PI3K-AKT (+)	Glycolysis (+)	71
	Adenovirus	E4-ORF1	PI3K-AKT (+)	?	67
	MNV	?	PI3K-AKT (+)	Glycolysis (+)	66
	EBV	LMP2A	PI3K-AKT-mTOR (+)	?	69 – 71
	HCV	Core protein (?)	PI3K-AKT (−)	Glycolysis (−)	73, 74
	IAV	?	PI3K-AKT (+)	Glycolysis (+)	63
AMPK signaling	HCMV	UL38?	CaMKK-AMPK (+)	Glycolysis (+)	76, 79, 80
	SV40	Small T antigen	AMPK (+)	Energy homeostasis	84
	VACV	?	AMPK (+)	?	86
	HCV	?	AMPK (−)	Lipid accumulation (+)	85
	HSV 1	?	AMPK [early (−), late (+)]	Lipid/protein synthesis (+) early, beta oxidation (+) late	83
Hypoxia-inducible factors	VACV	C16	HIF-1α (+)	Glutamine metabolism (+)	4, 41
	HPV 16, 18	E6, E7, E2	HIF-1α (+)	Glycolysis (+)	65, 94, 96
	HBV	HBx	HIF-1α (+)	Glycolysis (+) (?)	99 – 101
	EBV	LMP1, EBNA3, EBNA5	HIF-1α (+)	Glycolysis (+)	103 – 106
	KSHV	miRNA, GPCR, LANA (?)	HIF-1α (+)	Glycolysis (+)	109 – 111
	HCV	?	HIF-1α (+)	Glycolysis (+), OXPHOS (−)	113
	RSV	?	HIF-1α (+)	Glycolysis (+), PPP (+)	114
	IAV	?	HIF-1α (+)	Glycolysis (+) (?)	115, 116
	HIV	Vpr	HIF-1α (+)	Glycolysis (+)	117, 118
	HCMV	?	HIF-1α (+)	Kynurenine pathway (−)	119
Oncogenes and tumor suppressors	Adenovirus	E4ORF1	Myc (+)	Glycolysis (+), glutaminolysis (+)	68, 125
	Adenovirus	E1A, E1B	Myc (+), p53 (−)	?	126 – 128
	KSHV	?	Myc (+)	Glutaminolysis (+)	133
	KSHV	LANA	p53 (−)	?	137
	EBV	EBNA2	Myc (+)	One carbon metabolism (+), FA metabolism (+)	134 – 136
	EBV	BZLF1	p53 (−)	?	139
	HCV	NS3, NS5	p53 (−)	?	138
	HPV	E6	Myc (+), p53 (−)	Glycolysis (+)	129 – 131
Direct interaction of virus and host factors	HCMV	UL38 (?)	SREBP-2 (+)	Sterol biosynthesis (+)	143, 145
	HCMV	UL38 (?)	SREBP-1 (+)	FA biosynthesis (+)	144, 146
	DENV	NS3	FASN (+)	FA biosynthesis (+)	147
	DENV	NS1	GAPDH (+)	Glycolysis (+)	148

a Signaling pathways important for regulating cellular metabolism that are targeted by different viruses are shown. (+) indicates upregulation, (−) indicates downregulation, and (?) indicates unknown.

Because viruses are master manipulators of cell functions, studying virus-host interaction at the metabolic interface could reveal fundamental aspects of cellular metabolism. A better understanding of the basic mechanism involved in host-virus interactions could identify novel targets for developing therapeutic interventions for viral diseases and other pathologies. For example, metabolic alterations have emerged as common mechanisms that underlie the progression of cancers because cancers, like viruses, demand increased energy production and macromolecule biosynthesis to propagate.

## GROWTH FACTOR RECEPTOR SIGNALING

Among the major factors that govern cellular metabolism and proliferation are the receptor tyrosine kinase (RTK) pathways, particularly growth factor receptor (GFR) signaling. The GFR pathway determines whether a cell remains quiescent (metabolically inactive) or enters a state of active proliferation. Most terminally differentiated mammalian cells exist in a quiescent metabolic state, in which glucose is catabolized via glycolysis to produce pyruvate in the cytoplasm. Pyruvate is then transported to the mitochondria, where it is oxidized to CO_2_ via the TCA cycle. The NADH, NADH_2_, and FADH_2_ molecules produced during glycolysis and the TCA cycle are eventually used to drive ATP production via oxidative phosphorylation (OXPHOS) (9). Increased growth factor concentrations activate growth factor signaling pathways that enhance nutrient uptake, primarily glucose and glutamine (10), to support cell proliferation. The onset of cell cycle progression and proliferation increases the cellular demand for carbon, nitrogen, and other nutrients to generate carbohydrates, proteins, fats, nucleic acids, and energy (8). The uptake, synthesis, and breakdown of each biomolecule are further regulated by other signaling cascades, which will be discussed in more detail later. Constant activation of proliferative signaling and enhanced metabolic activity may result in the development of cancer (11), highlighting the importance of the tight regulation of cell signaling and metabolism.

Due to its crucial role in cell metabolism modulation, the GFR signaling pathway is targeted by several viruses to repurpose host metabolic pathways for their benefit (Fig. 1A and Table 1). Interestingly, some viruses encode growth factors that are homologous to those produced by the cells, allowing for the modulation of the RTK pathway (reviewed in references 12 and 13). One excellent example is the virus growth factor (VGF), the viral homolog of cellular epidermal growth factor (EGF), which is encoded by vaccinia virus (VACV), a prototypical poxvirus (14, 15). The deletion of VGF decreases VACV replication capacity in resting cells and mice (16, 17), highlighting the importance of this protein for the VACV life cycle. Furthermore, by inducing epidermal growth factor receptor (EGFR) and mitogen-activated protein kinase (MAPK) signaling, VGF can stimulate proliferative responses (18–20), and VGF is important for the motility of infected cells, which facilitates viral spread (21). VACV infection increases the demand for energy and macromolecules to support replication, and the induction of motility and proliferative responses also require additional energy (22, 23); therefore, VGF could represent a major factor involved in the induction of metabolic changes in VACV-infected cells. Remarkably, our global metabolic profiling of VACV-infected human foreskin fibroblasts (HFFs) showed that VACV infection increases the steady-state levels of several TCA cycle intermediates, including citrate (24). The deletion of VGF rendered VACV unable to enhance citrate levels, indicating that the elevation of citrate levels depends on VGF expression. Moreover, VACV infection stimulates the noncanonical phosphorylation of signal transducer and activator of transcription 3 (STAT3) at S727 in a VGF-dependent manner, and citrate upregulation requires the activities of EGFR, MAPK, and STAT3 signaling (24). Inhibition of any of these pathways severely impairs viral replication (24–26).

The upregulation of citrate levels by VGF-induced EGFR, MAPK, and STAT3 signaling (24) provides a mechanistic explanation for the observed alteration in metabolism, centered around the TCA cycle during VACV infection. VACV increases OXPHOS activity, as indicated by increased oxygen consumption rates (OCRs) and ATP levels (3, 27, 28). Although VACV induces the overall shutoff of host protein production (29), the translation of OXPHOS-related mRNAs is selectively upregulated by VACV infection (28). Additionally, VACV upregulates glutamine metabolism (2, 3, 30), and glutamine represents a critical carbon source for the TCA cycle. A study by Greseth and Traktman demonstrated that VACV depends on *de novo* FA synthesis to generate an energy-favorable environment (3), which suggests that VACV might modulate FA metabolism, which represents another major carbon source for the TCA cycle. While VGF is important for the upregulation of citrate levels, whether VGF expression is sufficient to induce the metabolic changes observed in VACV-infected cells remains unclear. The effects of VGF, which is secreted early during viral infection, on the modulation of other metabolic pathways are important to examine. In addition, further studies remain necessary to determine whether the EGF homologs encoded by other poxviruses are similarly involved in rewiring the host metabolism and whether the same cellular signaling cascades are involved in these processes. It is also of importance to determine the differential and shared effects of poxvirus growth factors and their cellular homologs.

Other forms of RTKs in the rewiring of cellular metabolism following viral infection could also be important. Baculovirus, which infects insects, encodes a fibroblast growth factor (FGF) (31) homologous to cellular FGF. The viral FGF, similar to VACV VGF, is essential for stimulating energy-consuming processes, such as cell migration (21, 32). Baculovirus infection upregulates several aspects of cellular metabolism, including increased glucose and glutamine consumption, increased amino acid metabolism, and increased oxygen consumption (33, 34). Although a subset of the FGF family of proteins (FGF 1, 15/19, and 21) are associated with regulation of metabolism (35), the exact role of viral FGFs in rewiring host cell metabolism is unknown. These studies provide the basis for studying the role played by baculovirus FGF in the modulation of host metabolism.

Although viruses such as VACV and baculovirus encode and secrete their own growth factors, other viruses appear to find alternative ways to stimulate growth factor signaling. Epstein-Barr virus (EBV), an oncogenic gammaherpesvirus, induces EGFR signaling via the latent viral membrane protein 1 (LMP1) (36). LMP1-mediated GFR signaling is essential for increasing glucose metabolism in EBV-infected cells (37). In human nasopharyngeal epithelial cells, LMP1 increases the uptake of glucose and glutamine, enhances the activity of lactate dehydrogenase A (LDHA), increases lactate production, and reduces the activity of pyruvate kinase and pyruvate concentration (37). Interestingly, the LMP1 protein of EBV also induces the activation of EGFR, extracellular signal-regulated kinase (ERK)-MAPK, and STAT3 phosphorylation (at both S727 and Y705) in human cervical carcinoma cell lines and rat primary fibroblasts (38). Further tests remain necessary to examine whether the metabolic changes induced by the EBV LMP1 protein are mediated by the activation of the EGFR-MAPK-STAT3 signaling axis and have metabolic effects comparable to VACV VGF, as well as whether these metabolic changes promote EBV’s oncogenesis.

Severe acute respiratory syndrome coronavirus (SARS-CoV) infection leads to the upregulation of EGFR ligands such as heparin-binding EGF-like growth factor (HB-EGF) and amphiregulin (AREG), and overactivation of EGFR leads to severe lung damage and pulmonary fibrosis (39). SARS-CoV-2, the causative agent underlying the current coronavirus disease 19 (COVID-19) pandemic, also induces growth factor signaling. A phosphoproteomics analysis of SARS-CoV-2 revealed the activation of GFR and downstream signaling molecules in infected cells (40). Notably, the SARS-CoV-2 infection increased the levels of several key enzymes associated with glycolysis, the TCA cycle, and central carbon metabolism (40, 41), which indicates the upregulation of these metabolic pathways at multiple levels. Furthermore, the inhibition of the GFR results in the severe suppression of SARS-CoV-2 replication (40), indicating a crucial role played by this pathway during COVID-19 progression. These findings suggest that SARS-CoV-2 infection may lead to metabolic diseases such as diabetes (42). Moreover, patients with metabolic disorders face a higher risk of infection with SARS-CoV-2 and are associated with significantly worse outcomes (43). Because of this bidirectional relationship, further studies examining the correlation between the GFR pathway and the metabolic alterations in SARS-CoV-2-infected cells could result in the identification of novel and effective therapeutic strategies against COVID-19.

Although the direct effects on metabolic alterations have not yet been established, many viruses are known to co-opt the GFR signaling pathway to promote various stages of viral replication, such as entry, internalization, and the subversion of antiviral responses (reviewed in reference 12). The dynamic interactions between the gene products of HCMV, UL135, and UL138 govern the attenuation or sustainment of EGFR signaling (44). Interestingly, UL138 is important for the induction of latency, and UL135 is essential for the reactivation of HCMV (45). Because a productive infection may be associated with different metabolic requirements than a latent infection, future studies that delineate the functions of these proteins and the EGFR pathway in metabolic changes could be used to identify novel approaches that can be applied to thwart HCMV infections. The E5 oncoprotein of human papillomavirus 16 (HPV16) stimulates the EGFR pathway and induces the prolonged activation of downstream MAPK and the Akt signaling pathway in response to EGF treatment (46). The E5 protein could indirectly modulate the Warburg effect, the switching of metabolism to aerobic glycolysis instead of OXPHOS for inefficient yet rapid production of ATP (47), on the HPV16-transformed cells via EGFR axis (48). The influenza A virus (IAV), hepatitis C virus (HCV), and hepatitis B virus (HBV) are examples of viruses that upregulate the EGFR pathway to increase virus uptake and internalization (49–51). Furthermore, the IAV- and rhinovirus-mediated activation of EGFR can dampen the interferon gamma-mediated antiviral responses, contributing to establishing a proviral environment (52). As discussed later in this article, infections with these viruses can lead to profound and specialized changes in cellular metabolism. Further studies that elucidate the role of the EGFR pathway or one of several signaling cascades downstream of EGFR could provide insights into the complex interactions between virus and host factors during the rewiring of cell metabolism, leading to the development of potent antiviral therapies.

## PI3K-Akt-mTOR PATHWAY

Because RTKs, such as GFRs, are activated upon the binding of membrane-localized receptors with extracellular ligands, they have the potential to govern the activation of several other metabolically important signaling cascades within a cell. The ubiquitous PI3K-Akt pathway is activated primarily by the binding of growth factors to extracellular receptors. Upon activation, PI3K recruits and activates other kinases, including Akt, to perform various functions (8). The activation of the PI3K-Akt axis results in increased glucose uptake, the stimulation of enzyme activity by several key glycolytic enzymes, and an increase in the overall glycolytic rate of the cell (reviewed in references 53). In addition to enhancing glycolysis, Akt promotes lipid metabolism through several mechanisms. First, Akt serves as an essential regulator of the enzyme ATP citrate lyase (ACLY) that converts the citrate transported out of mitochondria into acetyl coenzyme A (acetyl-CoA), a process necessary for lipid synthesis (54). Akt activation can cause indirect proteolytic release of sterol regulatory element-binding proteins (SREBPs) from the nucleus, leading to the induction of lipogenic genes essential for lipid metabolism (55). Mammalian target of rapamycin (mTOR) is another crucial regulator of cellular metabolism (reviewed in reference 56). mTOR is a key component of the multisubunit mTOR complex 1 (mTORC1) and mTOR complex 2 (mTORC2) protein complexes, which sense and regulate amino acid metabolism to control protein synthesis, cell growth, and proliferation. The growth factor-induced activation of the PI3K-Akt pathway can activate or relieve the inhibition of mTORC1 (56). Although mTORC1 acts downstream of Akt, mTORC2 acts upstream of Akt, widening the range of potential Akt substrates (57).

Because the PI3K-Akt-mTOR signaling pathway sits at the crossroads of several critical cellular pathways, many viruses have evolved multiple mechanisms to target this pathway (Fig. 1A) (reviewed in reference 58). Although the viral factors that interact with this pathway are known for some viruses, a considerable gap exists in how most viruses repurpose this cascade for metabolic reprogramming. The VACV-mediated activation of the PI3K-Akt pathway early during infection is important for viral replication and host cell survival (59). Lipid metabolism is essential for VACV to create an energy-rich state capable of supporting the increased demands during virus replication (3). We found that VACV infection increases the levels of carnitylated FAs necessary for β-oxidation (24). Furthermore, the acylation of several VACV proteins is essential for capsid envelopment and egress from the infected cell (60). VACV also depends on lipid rafts for entry into the cells (61), and integrin β-1 (a lipid raft-associated protein) plays a key role in virus endocytosis through the PI3K-Akt pathway (62). Further tests are required to determine the effects of VACV infection on fatty acid metabolism and identify viral factors and host cascades involved in the modulation.

Several other viruses have also been shown to modulate the PI3K-Akt-mTOR pathway (Fig. 1A and Table 1); however, similar to VACV, whether this modulation directly impacts metabolism remains incompletely understood. IAV has been shown to increases glycolysis, glucose uptake, and lactate excretion in a PI3K-Akt pathway-dependent manner (63). The inhibition of this pathway suppresses glycolysis and, subsequently, reduces IAV replication and increases survival in a mouse model (63). During which stage of IAV replication this metabolic regulation occurs and which viral proteins are responsible remain unknown. The E6/E7 proteins expressed by HPV16 are important for activating the PI3K-Akt pathway (64). The E6 oncoprotein could activate glycolysis through the activation of PI3K-Akt and mTOR pathways (48). The E7 protein is important for inducing glycolysis in HPV16-infected cells by binding and activating the glycolytic enzyme pyruvate kinase isozyme M2 (PKM2) (65). Murine norovirus-infected macrophages have been shown to upregulate glycolysis in the host cells by activating the Akt pathway, which is important during the early stages of viral replication (66). Adenovirus E4-ORF1 is vital for the activation of the PI3K-Akt pathway (67). While this activation is not responsible for inducing glycolysis in adenovirus-infected cells, the effects of this activation on a plethora of other metabolic changes that occur during adenovirus infection remain possible (68). The EBV latent membrane protein 2A (LMP2A) promotes the constitutive phosphorylation and activation of Akt via the PI3K-Akt pathway (69, 70), which leads to the activation of the mTOR pathway (71). Furthermore, LMP2A induces the expression of several transcription factors and genes associated with DNA/RNA metabolism and other metabolic enzymes (72), warranting further studies to elucidate the role of LMP2A in inducing metabolic alterations upon EBV infection.

Although some viruses activate the PI3K-Akt pathway to enhance glycolysis to support virus replication, others may do the opposite to ensure optimal survival, suggesting each virus may have evolved unique means to modulate signaling-metabolism cascade. HCV, most likely through the core protein, suppresses the PI3K-Akt pathway via the binding of tumor necrosis factor alpha, which inhibits the insulin receptor substrate and disrupts glucose metabolism by inhibiting glucose uptake via the downregulation of the glucose transporter 4 (GLUT4) and the upregulation of phosphoenolpyruvate carboxykinase 2 (PCK2) (73, 74). In addition to viruses that infect mammalian cells, white spot syndrome virus, an invertebrate virus that infects arthropod cells, induces glycolysis in a PI3K-Akt-mTOR dependent fashion (75). Targeting this cell signaling across species for metabolic alterations by a broad range of viruses again indicates the importance of metabolism reprogramming for diverse virus families.

## AMPK PATHWAY

Another essential signaling cascade of cellular metabolism is the AMPK pathway, which is sometimes referred to as the “metabolic master switch.” This pathway is activated to increase the AMP- or ADP-to-ATP ratio due to various physiological stresses or chemical inducers. Upon activation, this pathway leads to an increase in ATP synthesis via the activation of catabolic processes, such as the β-oxidation of FAs (76). Activated AMPK also triggers the destruction of existing defective mitochondria and enhances the synthesis of new mitochondria to support increased energy production during energy-deficient states. Through extensive cross talk with the growth factor-initiated PI3K-Akt pathway, the AMPK pathway also regulates the mTOR complex's activity to reprogram cellular metabolism (77). Because of the key role played by AMPK in the regulation of cellular metabolism, several viruses have evolved strategies to hijack AMPK signaling (Fig. 1B and Table 1) and remodel the host metabolome to promote efficient viral replication (78).

HCMV is among the best-studied models of virus-induced alterations to the host cell metabolism. HCMV induces profound changes in cellular metabolic pathways, including increased glycolysis, TCA cycle activity, glutamine metabolism, glutaminolysis, nucleotide metabolism, FA biosynthesis, and secondary metabolites and signaling molecules as prostaglandins (1). HCMV-induced AMPK phosphorylation and activation are central to the induction of these metabolic activation pathways, which result in a conducive environment for viral replication (79). The HCMV-mediated upregulation of AMPK results in increased glycolysis by enhancing glucose uptake via the upregulation of GLUT4 (79). The inhibition of AMPK not only reduces glycolytic flux but also suppresses HCMV replication. Interestingly, the HCMV-induced activation of AMPK leads to increased catabolism and reduced anabolism, limiting cell growth while simultaneously stimulating the overall metabolism (76). AMPK activation upon HCMV infection conditions is regulated by a calcium-calmodulin-dependent kinase kinase (CaMKK). One component that could be responsible for the activation of cellular AMPK is the viral immediate early protein UL38. The expression of this protein is both necessary and sufficient to promote glycolysis by activating glucose consumption and lactate excretion, glutamine consumption and glutamate secretion, and the secretion of proline and alanine (80). UL38 primarily binds to and inhibits the tuberous sclerosis protein complex 2 (TSC2) to activate the mTOR pathway, essential for maintaining a proviral environment (81). Interestingly, the activation of glycolysis via UL38 is dependent on its ability to inhibit TSC2 (80), suggesting interplay between this viral protein and the AMPK pathway. Additionally, HCMV proteins such as UL37, US28, US21, and UL146, which regulate calcium signaling, could indirectly activate the AMPK pathway due to significant overlaps in the downstream consequences of AMPK and calcium signaling (82).

The dynamic regulation of AMPK was observed during nerve cell infection with herpes simplex virus 1 (HSV-1) (83). Early after infection, HSV-1 downregulates the AMPK pathway to allow for the lipid and protein synthesis required for viral replication. This inhibition, however, gradually faded away after 4 h, coinciding with an increase in sirtuin 1 (SIRT1) expression. The maximum level of AMPK activation was observed later during the viral replication process. This increase in AMPK phosphorylation results in β-oxidation and mitochondrial biogenesis induction, which support productive viral replication (83).

Simian virus 40 (SV40), an oncogenic polyomavirus, activates AMPK pathways via its small T antigen to maintain energy homeostasis during glucose deprivation by inhibiting mTOR and activating apoptosis as an alternate energy source (84). Although AMPK acts as a proviral pathway, and most viruses tend to increase AMPK pathway activation, AMPK activation results in the suppression of HCV replication (85). While the viral proteins that regulate the suppression of AMPK during HCV infection remain to be elucidated, the loss of AMPK function appears to result in lipid accumulation in HCV-infected cells. Because the HCV life cycle heavily depends on cellular lipid levels, the accumulation of lipids could serve as a reservoir of the precursors required for HCV replication (85). In addition, AMPK has been found to play a broad role in regulating actin dynamics during VACV infection and is required to promote virus uptake (86). While so many viruses are affected by AMPK signaling, yet very little has been done to link the AMPK signaling and metabolic alterations during a viral infection, an area representing a significant knowledge gap.

## HYPOXIA-INDUCIBLE FACTOR (HIF) PATHWAY

Hypoxia-inducible factors (HIFs) are major regulators of cellular metabolism, especially during conditions of low oxygen availability (hypoxia) (87, 88). Because of the switch to anaerobic glycolysis, they promote glucose consumption and lactate excretion (89). By acting as transcriptional activators of several genes that promote the adaptation to hypoxic conditions, HIFs modulate key metabolic functions, such as FA, cholesterol, and mitochondrial metabolism (90), which are prime targets of viruses (Fig. 1C and Table 1).

One very well-studied example of virus-mediated HIF modulation is the VACV C16 protein-mediated stabilization of HIF-1α (4). VACV C16 binds directly and specifically to the human oxygen-sensing enzyme prolyl-hydroxylase domain-containing protein 2 (PHD2), inhibiting the PHD2-dependent hydroxylation of HIF-1α. The stabilization of HIF-1α by VACV results in a hypoxic environment, even under normoxic conditions. The infection with a recombinant virus lacking the C16 protein resulted in the decreased transcription of HIF-1α-responsive genes, such as vascular endothelial growth factor (VEGF), pyruvate dehydrogenase kinase 1 (PDK-1), and GLUT-1, which are important regulators of cellular metabolism (4). VACV-induced HIF-1α stabilization, however, did not increase lactate or a decrease in TCA cycle intermediates (30), which would be expected during a hypoxic response. In fact, we observed an increase in TCA cycle intermediates in VACV-infected cells (24), which appeared to be mediated, at least in part, by VGF-induced noncanonical STAT3 phosphorylation. The C16 protein of VACV, which is essential for HIF-1α activation, was necessary for the upregulation of glutamine metabolism (30). Several studies have shown that glutamine metabolism is critical for VACV replication either to feed the TCA cycle or to generate critical amino acids required for virus protein synthesis (2, 3, 91, 92). These studies elucidating the role played by the C16 protein in enhancing glutamine metabolism and the role played by VGF in the upregulation of TCA cycle metabolites have revealed new avenues for exploring other potential viral and cellular factors that might interact at the metabolic interface between VACV and its host. Signaling pathways mediated by EGF, the cellular homolog of VGF, are essential for HIF-1α stabilization via PI3K-Akt pathway activation in cancer cells (93). Therefore, the determination of whether VACV VGF induces metabolic alterations in a HIF-1α-dependent manner would be interesting to explore. How and whether the C16 protein and VGF might act together to rewire host cell metabolism in VACV-infected cells could be examined by generating a recombinant VACV that lacks both of these proteins if such a virus is viable.

HIF-1α activation is observed upon infection with several other viruses. The molecular mechanisms and metabolic consequences also have started to be elucidated. The E6 and E7 viral proteins of HPV induce HIF-1α activation, contributing to the observed induction of glycolysis following viral infection (94). The E6 protein attenuates interactions between the von Hippel-Lindau tumor suppressor (VHL) and HIF-1α, which induces the Warburg effect in HPV-infected cells. The HPV E7 protein, which enhances HIF-1α activity, directly interacts with the PKM2 enzyme that controls the exit of the glycolytic pathway, potentially diverting glycolytic intermediates toward anabolic metabolism (65, 95). The E2 protein of human papillomavirus HPV18 interacts with the mitochondrial membrane to induce the production of reactive oxygen species and induce glycolysis by activating HIF-1α (96).

HBV infection results in profound changes in cellular metabolism, affecting glycolysis, lipids, amino acids, vitamins, and nucleic acids. The HBV-induced increase in glycolysis has been attributed to at least three proteins: HBV core protein (HBc) (97); HBV pre-S2 mutant protein, which upregulates the expression and cell membrane localization of the glucose transporter GLUT4 (98); and the HBV X protein (HBx), which upregulates glucose-6-phosphate dehydrogenase (G6PD), as well as multiple other enzymes involved in gluconeogenesis (99, 100). HBx stabilizes HIF-1α (101). However, a direct association between the HBx-induced HIF-1α stabilization and alterations in cellular metabolism has not yet been explored. Because HBx is necessary for activating major metabolic pathways, such as the AMPK and mTORC1 pathways (102), the signaling cross talk between HIF-1α and other metabolic pathways is likely responsible for the altered metabolism observed in HBV-infected cells.

In addition to activating several key metabolic pathways, such as those associated with the FGFR, PI3K-Akt, and ERK-MAPK, the LMP1 protein of EBV enhances the degradation of PHD1 and PHD3 to activate HIF-1α (103), which upregulates glycolysis through the upregulation of PKM2 and PDH2 (104, 105). Moreover, LMP1 upregulates the key glycolytic enzyme hexokinase 2 (HK2) (106) and the glucose transporter GLUT1 (in an mTORC1-dependent manner) (107), contributing to enhanced glycolysis and indicating the existence of metabolic cross talk in signaling pathways induced by viral proteins. Another potential mechanism for the induction of glycolysis in EBV-infected cells is the viral proteins EBV nuclear antigen 3 (EBNA3)- and EBNA5-mediated stabilization of HIF-1α (108).

Another example of a virus that stabilizes HIF-1α to upregulate glycolysis is Kaposi’s sarcoma-associated herpesvirus (KSHV). KSHV infection upregulates HIF-1α and HIF-responsive glycolytic genes, such as PKM2, HK, GLUT1, and PDK1, through viral microRNAs (miRNAs), and G-protein-coupled receptors (GPCRs) (109–111). The latency-associated nuclear antigen (LANA), which is necessary for HIF-1α stabilization and nuclear translocation, and the induction of glucose transporter genes, such as GLUT1, is a candidate among several KSHV proteins that may mediate glycolysis in KSHV-transformed cells (112). Although the viral proteins responsible for this process have not been identified, HCV stabilizes HIF-1α, upregulating glycolytic enzymes and suppressing OXPHOS activities during HCV infection (113).

Other examples of virus-mediated HIF-1α stabilization and metabolic regulation include the respiratory syncytial virus (RSV) (114) and the H1N1 variant of IAV (115). A significant shift in metabolism toward glycolysis and the pentose phosphate pathway was observed during RSV infection in human small alveolar epithelial cells (114). The suppression of HIF-1α also reduced HK2, PDK1, and VEGF levels and reduced viral titers, suggesting an essential role of this pathway in upregulation in RSV replication (114). The H1N1 IAV variant increases GLUT1 levels by stabilizing HIF-1α via the inhibition of the proteasome and a decrease in factor inhibiting HIF-1 (FIH-1) expression (115). This mechanism could partially explain the observed increase in glycolysis, glucose uptake, and lactate excretion during early IAV infection (63, 116). Although the exact metabolic consequence remains unclear, the human immunodeficiency virus type 1 (HIV-1) viral protein Vpr induces stabilization of HIF-1α (117), resulting in the induction of two critical glycolytic enzymes, HK and PKM2 (118). In HCMV-infected cells, HIF-1α suppresses kynurenine levels and expression of the rate-limiting enzyme of kynurenine synthesis, indoleamine 2,3-dioxygenase 1 (119). HIF-1α reduces HCMV replication by regulating metabolism and metabolite signaling (119). Further studies remain necessary to identify viral factors required for HIF-1α induction during HCMV infection.

## ONCOGENES AND TUMOR SUPPRESSORS

Some viruses modulate host metabolism by interacting with cellular oncogenes (e.g., Myc) and tumor suppressors (e.g., p53) or introducing virus-specific oncogenes (7). Oncogenes and tumor suppressors are critical regulators of cellular metabolism (120). Mutations that result in the activation of KRas or Myc proteins may induce metabolically favorable environments for cell proliferation. The activation of these oncogenes can induce glycolysis, OXPHOS, pentose phosphate pathways, and lactate production. Moreover, KRas and Myc activation result in increased glutamine uptake to feed the TCA cycle for energy production, a process known as glutaminolysis (121). In addition to activating key lipid metabolism enzymes and nucleotide biosynthesis, Myc is involved in the biogenesis of organelles, such as mitochondria and ribosomes (121, 122). Tumor suppressors, including but not limited to p53, phosphatase and tensin homolog (PTEN), SIRT3, and SIRT6, act on several stages of metabolism such as glycolysis, TCA cycle, and lipid metabolism (7). The role played by p53 in metabolism has been extensively studied. Wild-type (WT) p53 protein activates mitochondrial metabolism and lipid catabolism, suppressing glycolysis and lipid synthesis, whereas the gain of function mutation in p53 leads to the exact opposite functions (123, 124).

An excellent example of the virus-induced activation of the Myc oncogene has been observed in adenovirus-infected cells. A nontumorigenic breast epithelial cell line infection with adenovirus 5 (Ad5) strain induced glycolytic metabolism indicated by increased glucose consumption and lactate production and decreased oxygen consumption (68). The E4ORF1 protein of Ad5 is sufficient to increase glucose metabolism in infected cells, binding to and activating Myc to increase the transcription of key glycolytic enzymes, including HK2 and phosphofructokinase (PFK) (68). The E4ORF1-mediated activation of Myc is not only important for the activation of glycolysis but also for altering glutamine metabolism (125). Myc activation is necessary for the Ad5-induced increase in glutamine utilization, including the increased expression of glutamine transporters and glutaminolysis enzymes, such as glutaminase (GLS) (125). However, the observed decrease in the OCR following Ad5 infection is independent of the E4ORF1-induced activation of Myc (68). Interestingly, the adenovirus E1A protein is involved in Myc activation (126). Furthermore, E1A and E1B 55K can induce the suppression of the tumor suppressor p53 (127). Although the metabolic consequences of these changes remain largely unknown, these findings suggest that the E1A and E1B proteins could account for altered cellular metabolism during adenovirus infection (128).

The E6 oncoprotein of HPV16 interacts with c-Myc proto-oncogene, promoting glycolytic genes such as HK2, LDHA, and PFK (129, 130). HPV E6, through its association with E6-associated protein, an E3 ubiquitin ligase, induces the degradation of p53 (131). Because p53 is known to inhibit glycolysis and promote OXPHOS (132), the overall activation of glycolysis observed during HPV infection could, at least partially, be attributed to E6 and its regulation of c-Myc and p53.

Other viruses that interact with oncogenes or tumor suppressors to increase replication efficiency include KSHV, EBV, HPV, and HCV (Fig. 1D and Table 1). The latent KSHV infection of endothelial cells induces the expression and upregulation of Myc, and the targets of Myc, including the glutamine transporter SLC1A5, which could explain the upregulation of glutamine uptake and glutamine addiction during KSHV infection (133). The interaction of EBV nuclear antigen 2 (EBNA2) with its target c-Myc results in the oncogenic transformation of primary B lymphocytes by initiating the cell cycle entry in resting B cells (134). Interestingly, EBNA2 and Myc are required for induction of aerobic glycolysis and the upregulation of mitochondrial one carbon (1C) metabolism (135). As the master regulator of B cell metabolic reprogramming during EBV infection, EBNA2-induced 1C metabolism drives the production of nucleotides, glutathione, and mitochondrial NADPH (135). EBNA2, Myc, and SREBP are also essential for inducing cholesterol and FA biosynthesis by inducing key enzymes such as acetyl-CoA carboxylase 1 (ACC1) and FA synthase (FASN) (136). These findings highlight the role of viral remodeling of host signaling and metabolic changes in cancer development during EBV infection. The latent viral proteins LANA expressed by KSHV, basic leucine zipper nuclear factor 1 (BZLF1) expressed by EBV, E6 expressed by HPV, and NS3 and NS5 expressed by HCV all downregulate the tumor suppressor p53 (137–140). The impact of this suppression on the changes observed in host cell metabolism during infections remains unclear.

## OTHER MECHANISMS

In addition to altering signaling pathways, cellular metabolism could be regulated through feedback activation/inhibition mechanisms mediated by various metabolites and by-products (141). Several key enzymes and metabolic regulators, such as carbohydrate response element-binding protein (ChREBP) and SREBP, could exert regulatory functions on the metabolic activities in cells (142). In addition, viruses likely have evolved mechanisms to directly interact with the key metabolic enzymes and regulators found in host cells (Table 1).

HCMV infection upregulates ACC1 levels, the rate-limiting enzyme involved in FA biosynthesis (143, 144), through two separate mechanisms. First, HCMV infection results in the activation of SREBP-2 (143), the master regulator of sterol biosynthesis (145), which activates ACC1 in an mTORC1 activation-dependent manner (143). Second, HCMV induces lipogenesis through the proteolytic cleavage and activation of SREBP-1, a major regulator of FA biosynthesis (146), to activate ACC1 (144). The viral protein UL38, which interacts with and inhibits TSC1/2 to activate mTORC1 (81), could also play a key role in the modulation of FA metabolism in HCMV-infected cells.

Another case of interaction between viral and host metabolic factors is the interaction between the dengue virus (DENV) nonstructural protein 3 (NS3) and the host cell FA synthase (FASN), a key enzyme necessary for FA biosynthesis (147). By binding to FASN, NS3 relocates the host enzyme to the virus replication site, increasing the rate of FA biosynthesis (147). Another example is the direct interaction between the DENV NS1 and the cellular glycolytic enzyme glyceraldehyde-3-phosphate dehydrogenase (GAPDH) (148). Interestingly, DENV infection requires glycolysis for optimal replication (149) and induces glycolytic processes by upregulating the levels of GLUT1 and HK (149). The binding between DENV NS1 and GAPDH increases GAPDH activity (148), representing one example of an interaction between a viral and host factor that elevates glycolysis. Mechanistic studies that identify these interactions can provide a platform for developing therapeutics against the viral pathogen.

## CONCLUDING REMARKS, LIMITATIONS OF THE CURRENT STUDIES, AND OUTLOOK FOR THE FUTURE

In summary, virus-induced metabolic alterations can significantly affect the progression of viral infection. The central theme of these alterations suggests that interactions between one or more viral factors and various cellular signaling pathways or factors can lead to metabolic reprogramming. Although some of the key players involved in the virus-mediated hijacking of cellular metabolism have been identified, many remain unknown. In some cases, a single viral protein can regulate several different host factors, whereas in other cases, similar effects require multiple proteins. Extensive cross talk occurs among various signaling pathways that govern metabolism, posing the unique challenge of carefully teasing apart the roles of viral factors in repurposing cellular metabolism.

Although we attempt to review the mechanisms through which viruses hijack cellular signaling to rewire host metabolism, there is still a striking lack of mechanistic studies in this field. Many studies have conferred the functions of viral proteins to key metabolic regulators without describing specific metabolic phenotypes. Conversely, several viruses have been identified to alter key signaling pathways related to metabolism, but these alterations in host metabolism have not been elucidated. Some viral factors may have evolved unique means to manipulate signaling pathways by which they exert different effects on metabolism compared to cells without viral infections. A deeper understanding of metabolic signaling remains crucial for developing therapeutics that target metabolic pathways in viral infections. Additional studies remain necessary to identify viral and host factors that interact at the metabolic interface.

Although viruses are known to induce metabolic perturbations and these changes can shape viral replication and infection outcomes, the exact stages of replication for which these metabolic pathways are important have not been elucidated for many viruses. Alternatively, because the host provides the resources and precursors necessary for viral replication, the effects of the host’s metabolic status on viral replication could represent a fertile ground for future research. A better understanding of the dependence of specific viral replication stages on certain metabolic pathways will clarify why viruses target distinct metabolic aspects. Similarly, by understanding the effects of the host metabolic status on virus replication, mediators of viral tropism could be identified by whether some cells are more susceptible to viral infection than others. Susceptibility differences could also explain discrepancies observed in the metabolic reprogramming profiles of different cells following infection with the same virus.

Another major limitation of current studies is that most explorations of virus-host cell metabolism are performed in cultured cells, which may differ significantly from metabolic profiles in animal tissues. Most cells in an animal model are quiescent (150), with reduced metabolic activity (151), in contrast with the proliferating cells used in most studies (5, 6). Different tissues and organs might also have different metabolic statuses that might affect tropism and viral infectivity. Furthermore, animal metabolism is governed by the host’s diet and immune system activity, and the effects of these regulations represent significantly understudied areas in the field of virus-host interaction at the metabolism interface. Viruses may target metabolism to alter a cell’s immune status or evade immune clearance (152, 153). Conversely, viral infection-induced metabolic alterations could trigger immune activation or the activation of antiviral pathways. Some immune cells detect viral metabolites and pathways altered by viruses (6, 154), adding layers of complexity. For some viruses such as EBV, there is a lack of an *in vivo* model for infection because it does not typically infect commonly used murine models (155, 156). The humanized mice that have reconstituted human immune system components (155–159) could be beneficial for the study of host-virus interactions at the metabolic interface. Additional *in vivo* studies examining the effects of virus-induced alterations in metabolism are necessary to better understand viral metabolism and the potential development of effective antiviral therapeutics.

Most current studies examining alterations in metabolic processes upon viral infection focus on steady-state metabolite levels. Because metabolism is a dynamic process that can involve rapid changes in the uptake, synthesis, and degradation of biomolecules, observed increases in the levels of specific metabolites could result in multiple interpretations that indicate various, sometimes opposing outcomes. For example, an increase in the steady-state levels of any given metabolite could indicate either increased synthesis or reduced consumption. Metabolomics should be coupled with studies that define the ongoing metabolic activities to overcome these challenges. A better understanding of the interactions between viruses and host metabolism can be achieved through the careful design and rigorous interpretation of metabolic flux profiling. Coupling these studies with studies examining the activation or suppression of enzymatic activities using chemical and genetic approaches could provide more comprehensive pictures of the metabolic landscapes of virus-infected cells.

Other aspects that should be considered during the study of virus-host interaction at the metabolic interface include selection of cell lines and the growth and nutrient conditions. While in some studies, cancer cells are desired research objects, such as viral oncogenesis and oncolytic viral therapy, in many other studies, the use of transformed cells should be avoided because the metabolism of cancer cells has already been altered compared to the metabolism of the primary cell lines. In addition, caution should be practiced while designing and interpreting *in vitro* metabolic studies because the cell culture media used provides nutrient-rich conditions that may not truly reflect the metabolic profiles of the natural host.

The studies of virus-induced reprogramming of host metabolism can facilitate fundamentally understanding mechanisms of cellular metabolic regulation. Importantly, the knowledge gained could be used to combat nonviral diseases, including cancers and many metabolic disorders. It is well established that cancer cells have dramatically altered metabolism. Studies examining cancer cell metabolism have greatly facilitated our understanding of cell metabolism in response to viral infections. Although several viruses have been found to interact with and alter tumor suppressors and oncogenes during rewiring cellular metabolism, whether these metabolic perturbations lead to the transformation of infected cells in a manner that promotes cancer development remains unclear. A better understanding of virus-induced changes in cell signaling that result in metabolic reprogramming may identify fundamental mechanisms involved in regulating cellular metabolism and the metabolic regulation that occurs in cancer cells. In addition to cancers, the study of virus-host interactions at the metabolic interface could also shed light on the progression of various metabolic disorders, such as obesity, dyslipidemia, and increased glucose levels. Metabolic disorders may increase the risk of certain viral diseases, including influenza and coronaviruses (160). Patients with metabolic diseases such as obesity and diabetes are at a higher risk of infection with SARS-CoV-2 and are associated with significantly worse outcomes of COVID-19 (43). On the other hand, viruses such as SARS-CoV-2, HCV, and HIV may induce metabolic disorders (41, 42, 161). Additionally, metabolic disorders may impair the host’s immunological response, facilitating viral infections that can worsen the severity of metabolic disorders (160). The study of virus-induced metabolic alterations might also provide avenues for developing novel strategies to combat metabolic disorders.

Overall, the study of virus-host metabolism can unquestionably facilitate the identification of various therapeutic windows associated with the viral dependence on specific enzymes or nutrients, which can be utilized to develop novel therapies against viral diseases and other metabolism-associated pathologies, including cancers.
